# Clinical benefit of midodrine hydrochloride in symptomatic orthostatic hypotension: a phase 4, double-blind, placebo-controlled, randomized, tilt-table study

**DOI:** 10.1007/s10286-016-0363-9

**Published:** 2016-07-02

**Authors:** William Smith, Hong Wan, David Much, Antoine G. Robinson, Patrick Martin

**Affiliations:** Volunteer Research Group, University of Tennessee Medical Center, Knoxville, TN USA; Shire, Lexington, MA USA; Barclay Consulting LLC, Ardmore, PA USA; Global Clinical Pharmacology & Pharmacokinetics, Shire, 300 Shire Way, Lexington, MA 02421 USA

**Keywords:** Midodrine, Orthostatic hypotension, Clinical trial

## Abstract

**Objective:**

Midodrine hydrochloride is a short-acting pressor agent that raises blood pressure in the upright position in patients with orthostatic hypotension. The US Food and Drug Administration’s Subpart H approval, under which midodrine was initially approved, requires post-marketing studies to confirm midodrine’s clinical benefit in this indication. The purpose of this study was to evaluate the clinical benefit of midodrine with regard to symptom response.

**Methods:**

This was a double-blind, placebo-controlled, randomized, crossover, multicenter study (NCT01518946). Following screening, patients aged ≥18 years with severe symptomatic orthostatic hypotension and on a stable dose of midodrine for at least 3 months were randomized to treatment with either their previous midodrine dose or placebo on day 1 and the respective alternate treatment on day 2. The primary endpoint measured time to syncopal symptoms or near-syncope using a 45-min tilt-table test at 1 h post-dose.

**Results:**

Thirty-three patients were screened for inclusion: 19 received at least one dose of midodrine and had at least one post-dose measurement of the primary endpoint. The least-squares mean time to syncopal symptoms or near-syncope after tilt-table initiation (mean ± standard error) was 1626.6 ± 186.8 s for midodrine and 1105.6 ± 186.8 s for placebo (difference, 521.0 s; 95 % confidence interval 124.2–971.7 s; *p* = 0.0131). There were 15 adverse events in 10 patients; all of these were mild or moderate in severity, with none considered by the investigators to be related to midodrine.

**Interpretation:**

Midodrine is a well-tolerated and clinically effective treatment for symptomatic orthostatic hypotension.

## Introduction

Patients with orthostatic hypotension experience a reduction in blood pressure when they stand up, which can result in clinical symptoms of dizziness, blurring of vision, fainting, and falls [1, 2]. The condition can have a substantial impact on health-related quality of life [1, 3]. Several therapies have been used for its treatment, including fludrocortisone, methylphenidate, ephedrine, indomethacin, and dihydroergotamine [1, 4]. However, none of these agents are indicated for the condition, and they are also associated with various adverse events. Droxidopa, a prodrug metabolized to norepinephrine, was approved by the US Food and Drug Administration (FDA) in 2014 for the treatment of orthostatic dizziness, lightheadedness, or near-syncopal feelings in adult patients with symptomatic neurogenic orthostatic hypotension, but its effectiveness has not been established beyond 2 weeks of treatment [5].

Midodrine hydrochloride, a prodrug for the active metabolite desglymidodrine, received conditional approval in the USA in 1996 for the treatment of orthostatic hypotension [6]. Desglymidodrine is an alpha-1 agonist that increases blood pressure and vascular tone via stimulation of arterial and venous alpha-adrenergic receptors [6]. It does not stimulate cardiac beta-adrenergic receptors and, owing to its poor diffusion across the blood–brain barrier, generally has no effect on the central nervous system [6]. The pressor effects of midodrine occur within approximately 1 h of the oral administration of a single dose, and the effects usually persist for about 4 h [6, 7].

Studies conducted in the USA [3, 8] and elsewhere [6, 9, 10], which included over 4000 patients, have shown midodrine HCl to be of value in the treatment of orthostatic hypotension. The FDA’s accelerated Subpart H approval of midodrine in 1996 considered a decrease in the frequency of the reductions in blood pressure that occur after standing in patients with orthostatic hypotension to be a “surrogate marker of effectiveness” that would likely “correspond to a clinical benefit”. Midodrine has been shown to increase blood pressure and vascular tone and to reduce the frequency and severity of syncopal symptoms in patients with orthostatic hypotension [11, 12], but full approval of the drug requires post-marketing studies to confirm that midodrine provides a clinical benefit for patients with symptomatic orthostatic hypotension [2, 13].

The objective of this phase 4 study was to assess the effect of midodrine on symptom response in the form of time to onset of syncopal symptoms or near-syncope measured using a protocol-defined tilt-table test at 1 h post-dose.

## Methods

### Study overview and participants

This was a double-blind, placebo-controlled, randomized, crossover, multicenter study conducted at six sites in the USA from 14 May 2012 to 22 June 2013. The study was carried out in an inpatient setting. Men and women aged 18 years or older who had a documented history of severe symptomatic orthostatic hypotension (e.g., due to Parkinson’s disease, Shy–Drager syndrome, multiple system atrophy, pure autonomic failure, or autonomic neuropathies) were eligible for enrollment. To be eligible for inclusion, individuals had to have been on a stable dose of midodrine for at least 3 months, been ambulatory when receiving adequate therapy for their symptomatic orthostatic hypotension, and had at least one of the following symptoms while standing or when not on treatment: dizziness, lightheadedness, feeling faint, or feeling like they might lose consciousness.

Additional eligibility criteria were: women of child-bearing potential must have had a negative serum beta human chorionic gonadotropin pregnancy test and must have abstained from sexual activity that could have resulted in a pregnancy or had agreed to use acceptable contraceptives throughout the period of the entire study and for 30 days after the last dose of midodrine; willingness and ability to undergo the procedures required by the protocol, including inpatient stay as required; adequate hydration status (as assessed by physical examination and clinical laboratory parameters, e.g., urine specific gravity); and ability to provide written, signed, and dated informed consent to participate in the study. Patients who had completed a previous midodrine study (SPD426-405; NCT01515865), which had the same entry criteria and assessments of the severity of symptomatic orthostatic hypotension as the current study, could enter the randomized phase of this study within 28 days of discharge from SPD426-405 without repeating screening assessments or within 2 months without repeating symptom severity assessments.

Patients were not eligible to participate in the study if they: were pregnant or lactating; had pre-existing sustained supine hypertension (two measurements at least 5 min apart with the patient continuously supine and at rest) greater than the drug label-recommended level (systolic blood pressure >180 mmHg, diastolic blood pressure >110 mmHg) or had these measurements at the screening visit; were using other medications, unless approved by the study physician; had a clinically significant clinical laboratory test abnormality during screening; had participated in other studies of investigational drugs or devices in the 30 days before enrollment in this study (other than study SPD426-405); had current or relevant history of physical or psychiatric illness, any medical disorder that may have required treatment or made the patient unlikely to comply fully with the requirements of the study or to complete the study, or any condition that presented undue risk from midodrine or study procedures; had a concurrent chronic or acute illness, disability, or other condition (including significant unexpected laboratory or ECG findings) that might have confounded the results of the tests and/or measurements administered in this study, or that might have increased the risk to the patient; had known or suspected intolerance or hypersensitivity to midodrine, closely-related compounds, or any of the stated ingredients; had a prior enrollment failure or randomization in this study; or had a history of alcohol abuse or other substance abuse within the last year.

### Study design

**Fig. 1:**
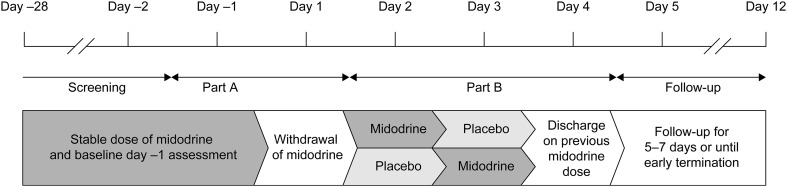
Study design. This was a double-blind, placebo-controlled, randomized, crossover, multicenter study. After an open-label screening period of 28 days, during which patients continued their usual, pre-study midodrine dose, baseline assessments of the severity of symptomatic orthostatic hypotension were undertaken on day −1. Midodrine treatment was withdrawn on day 1 (*Part A*) after which eligible participants entered the double-blind, randomized, crossover period (*Part B*). Patients were discharged on day 4, and their previous midodrine dose was reinstated. They were then followed up for 5–7 days

The study began with an open-label screening period of 28 days, during which patients continued their usual, pre-study midodrine dose (Fig. 1). Baseline assessments of the severity of symptomatic orthostatic hypotension were undertaken on day −1, and midodrine treatment was withdrawn on day 1 (Part A). Participants were eligible to enter the double-blind, randomized, crossover period (Part B) if the following criteria were met: (1) increase of at least 4 points in Orthostatic Hypotension Symptom Assessment Item 1 (OHSA) questionnaire [14] score between day −1 and day 1; (2) no syncopal or near-syncopal event or, at most, mild orthostatic symptoms within 15 min after transitioning from supine to standing on day −1 and syncope or a near-syncopal event or more numerous or more severe orthostatic symptoms on day 1; and (3) decreases in standing systolic blood pressure of at least 20 mmHg and standing diastolic blood pressure of at least 10 mmHg within 15 min after transitioning from the supine to the standing position on day 1. Physicians and investigators assessed the patients’ physical and mental wellbeing and ensured that patients were able to complete the OHSA questionnaires on both days.

Eligible patients were randomized for Part B in a 1:1 ratio to one of two treatment sequences: midodrine HCl followed by placebo, or placebo followed by midodrine HCl. The randomization number assigned to each patient was obtained from interactive response technology, which was managed by an external vendor (Bracket, Langhorne, PA). Randomization typically occurred after qualification on day 1, and the first randomized dose was administered on day 2.

During the double-blind phase (Part B), patients were treated with midodrine or placebo on day 2, according to their previous dose, and underwent a tilt-table test at 1 h post-dose. On day 3, they were given the respective alternate treatment and again underwent a tilt-table test at 1 h post-dose. Patients were discharged on day 4, and their previous midodrine dose was reinstated. They were then followed up for 5–7 days.

The study protocol, any protocol amendments, the final approved informed consent document, relevant supporting information, and all types of patient recruitment information were submitted and approved by site-specific institutional review boards and regulatory agencies (as appropriate) prior to study initiation. The study was conducted in accordance with the International Conference on Harmonisation of Good Clinical Practice and the principles of the Declaration of Helsinki, as well as other applicable local ethical and legal requirements. Patients provided written informed consent before taking part in any study-specific procedures.

### Tilt-table test procedure

At 1 h post-dose, participants lay supine on a tilt table for 30 min. The table was then tilted from 0 degrees (horizontal) to 90 degrees (vertical, with head up) in 30 s and was maintained in that position for 45 min or until the primary endpoint was reached. Time to endpoint was recorded. If no event occurred within 45 min, 2700 s was recorded as the time to primary endpoint. To ensure blinding, although institutional safety protocols may have required the assessment of vital signs, blood pressure measurements were not available to the blinded study staff, and orthostatic blood pressure data were not used to evaluate the primary endpoint.

### Endpoints

The primary endpoint was the time to onset of syncopal symptoms or near-syncope (participants felt sufficiently dizzy, lightheaded, faint, or as if they were about to lose consciousness so that they requested the table to be returned to horizontal, or they looked to be about to lose consciousness based on investigator assessment) during the tilt-table test.

Safety assessments included adverse events, vital signs, clinical laboratory parameters, electrocardiograms (ECGs), and physical examination.

### Analysis sets

**Fig. 2:**
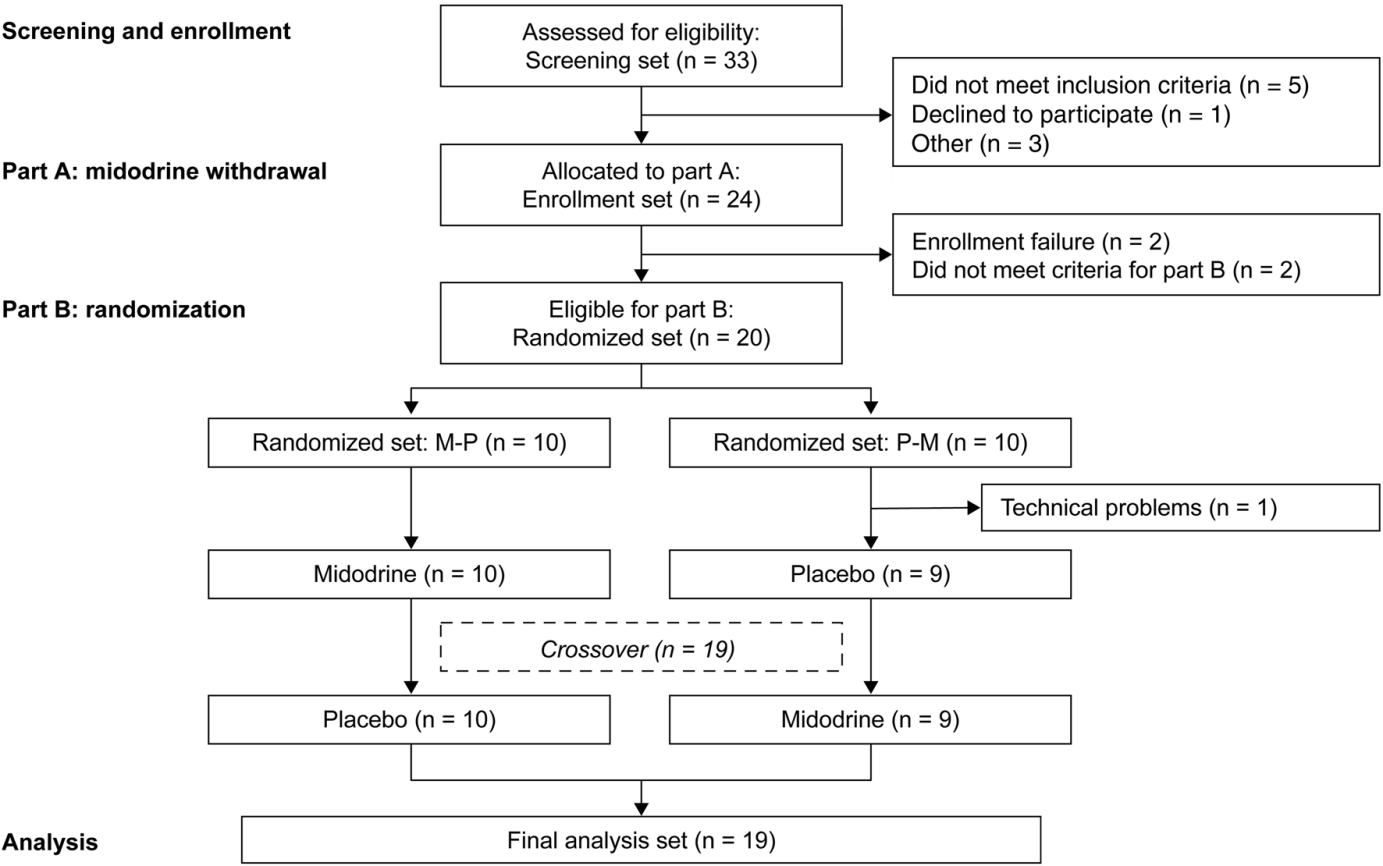
Participant flow. Thirty-three patients were screened for inclusion in the study and participated in the assessments on day −1 and day 1; of these, nine did not meet the inclusion criteria. The *enrollment set* comprised 24 participants. Four patients were withdrawn from the study during *Part A*. The *randomized set* comprised the 20 participants who received at least one dose of midodrine in *Part B*. One participant did not complete the study because of technical problems with the tilt-table on day 2 and was excluded from the *final analysis set*

Three analysis sets were defined for use in this study (Fig. 2). The *enrollment set* comprised all patients enrolled in the study who participated in assessments on day −1 and day 1. The *randomized set* comprised all patients enrolled in the study who received at least one dose of midodrine in Part B. The *final analysis set* comprised all patients enrolled in the study, who received at least one dose of midodrine and had at least one post-dose measurement of the primary endpoint.

### Sample size determination and statistical analysis

Assuming a standard deviation of 240 s for the within-patient difference in time to onset of syncopal symptoms or near-syncope, a power level of 80 % and a significance level of 0.05 (two-sided), it was calculated that the primary endpoint would need to be assessed in approximately 18 patients (9 patients in each treatment sequence) to detect a treatment difference of 180 s between treatments.

Summary statistics for the within-patient differences between treatments (midodrine minus placebo) in time to onset of syncopal symptoms or near-syncope were calculated. A Shapiro–Wilk test for normality was applied to the within-patient differences, and the resulting *p* value determined whether analysis of variance (ANOVA) or a non-parametric procedure, i.e., the Wilcoxon signed-rank test as suggested by Koch [15], should be used for the primary analysis.

For the ANOVA procedure, least-squares mean, standard error, difference in LS mean (midodrine HCl–placebo) and *p* value were based on type III sum of squares from an ANOVA model for time to onset of syncopal symptoms/near-syncopal symptoms, including treatment sequence (two levels), treatment (two levels), and treatment period (two levels) as fixed effects and subject-within-sequence as a random effect.

All analyses were performed using SAS^®^ version 9.1.3 or higher (SAS Institute, Cary, NC, USA).

## Results

### Study enrollment

In total, 33 patients were screened for inclusion in the study; of these, nine did not meet the inclusion criteria. Therefore, the enrollment set comprised 24 participants, of whom seven had previously completed study SPD426-405. Four patients were withdrawn from the study during Part A due to enrollment failure (two patients) or not meeting criteria for continuation to Part B (two patients). Therefore, the randomized set comprised 20 participants. One participant did not complete the study because of technical problems with the tilt-table on day 2 and was excluded from the final analysis set.

### Patient characteristics and demographics

The 19 patients in the final analysis set had a mean age of 43.5 years (range 18–78 years, Table 1), with 42.1 % under 40 years and 42.1 % aged between 40 and 65 years. Patients were predominantly female (94.7 %) and white (84.2 %), and had a mean body mass index of 26.7 kg/m^2^ [standard deviation (SD): 6.1; Table 1]. Demographics were similar in the randomized set (data not shown). Patients’ first daily dose of midodrine ranged from 2.5 mg to 15 mg.

**Table 1:** Study participant characteristics in the final analysis set

Characteristics	Total (*N* = 19)
Age (years, range)	43.5 ± 17.9 (18–78)
Sex	
Men	1 (5.3)
Women	18 (94.7)
Race	
White	16 (84.2)
Black	2 (10.5)
Native American or Native Alaskan	1 (5.3)
Body mass index (kg/m^2^)	26.7 ± 6.1

Pre-trial midodrine dose (mg)	Total (*N* = 19)
2.5	1 (5.3)
5.0	8 (42.1)
10.0	9 (47.3)
>10.0	1 (5.3)

Diagnostic history^a^	Total (*N* = 14)
Cancer	3 (21.4)
Depression	5 (35.7)
Diabetes/insulin resistance	2 (14.3)
Ehlers–Danlos syndrome	6 (42.9)
Gastroesophageal reflux disease	4 (28.6)
Gastroparesis	5 (35.7)
Hypothyroidism/thyroidectomy	8 (57.1)
Parkinson	2 (14.3)

Data are presented as mean ± standard deviation or *n* (%)

^a^Diagnostic history not recorded for all patients

### Supine and orthostatic blood pressure

Blood pressure was recorded during part A of the study on days−1 (with midodrine) and 1 (without midodrine) (Table 2). High (>160 mmHg) supine systolic blood pressure was recorded in two patients on day −1 and in one of these patients on day 1. One patient experienced high (>100 mmHg) supine diastolic blood pressure on day −1. No incidences of supine hypertension were recorded during Part B of the study, and blood pressure measurements were not assessed during the tilt-table test to ensure that blinding was maintained.

**Table 2:** Supine and orthostatic blood pressure with (day −1) and without (day 1) midodrine in the randomized set

	With midodrine^a^			Without midodrine		
	*n*	Systolic (mmHg)	Diastolic (mmHg)	*n*	Systolic (mmHg)	Diastolic (mmHg)
Supine orthostatic	20	119.4 ± 22.19	72.4 ± 12.91	20	116.5 ± 16.35	71.7 ± 13.97
3 min	20	106.8 ± 20.89	70.0 ± 15.57	18	100.6 ± 14.87	68.1 ± 14.07
5 min	19	107.7 ± 19.93	70.7 ± 15.53	16	95.0 ± 17.10	62.6 ± 14.71
7 min	18	105.1 ± 16.15	72.1 ± 14.22	10	94.9 ± 12.44	62.6 ± 12.00
9 min	17	103.8 ± 19.97	72.6 ± 15.97	9	89.6 ± 14.00	56.3 ± 9.17
11 min	16	106.8 ± 19.43	74.1 ± 16.30	5	94.8 ± 13.01	57.2 ± 5.17
13 min	16	104.1 ± 18.62	73.8 ± 14.90	3	91.3 ± 7.02	57.3 ± 5.03
15 min	16	103.9 ± 18.78	73.9 ± 15.12			

Data are presented as mean ± standard deviation

^a^Patients’ own midodrine

### Primary endpoint

The least-squares (LS) mean time to onset of syncopal symptoms or near-syncope (mean ± standard error [SE]) was 1626.6 ± 186.8 s after initiation of the tilt-table test in patients receiving midodrine and 1105.6 ± 186.8 s in patients receiving placebo (Table 3). The Shapiro–Wilk test for normality was applied to the within-patient difference between treatments (midodrine HCl–placebo). The resulting *p* value of 0.2672 confirmed the normal distribution of the patient population, and the ANOVA model was used as described for subsequent analysis. No statistically significant differences were related to the treatment sequence (*p* = 0.5035) or the treatment period (*p* = 0.9811).

**Table 3:** Analysis of within-patient differences in time to onset of syncopal symptoms or near-syncope after initiation of a tilt-table test in patients receiving midodrine or placebo in the final analysis set

	Placebo	Midodrine
Day 2 (*N*)	9	10
Completed tilt-table test, *n*/*N* (%)	1/9 (11.1)	3/10 (30.0)
Time to onset (seconds)^a^		
Mean ± SE	1218.2 ± 253.15	1518.5 ± 314.94
Median (95 % CI)	1136.0 (634.5–1802.0)	1279.0 (806.1–2230.9)
Range	252–2700^b^	299–2700^b^
Day 3 (*N*)	10	9
Completed tilt-table test *n*/*N* (%)	0/10 (0)	3/9 (33.3)
Time to onset (seconds)^a^		
Mean ± SE	993.0 ± 162.82	1734.7 ± 301.80
Median (95 % CI)	1163.0 (624.7–1361.3)	1870.0 (1038.7–2430.6)
Range	198–1690	455–2700^b^
Overall time to onset (seconds)^a^		
*n*	19	19
Mean ± SE	1099.7 ± 145.50	1620.9 ± 214.30
Median (95 % CI)	1136.0 (794.0–1405.4)	1560.0 (1170.7–2071.1)
LS Mean ± SE^c^	**1105.6** **±** **186.82**	**1626.6** **±** **186.82**
Difference in LS Mean (95 % CI)		521.0 (124.2–917.7)
Sequence effect *p* value^c^		0.5035
Period effect *p* value^c^		0.9811
Treatment effect *p* value^c^		**0.0131**

Bold values indicate the primary endpoint

*ANOVA* analysis of variance, *CI* confidence interval, *HCl* hydrochloride, *SD* standard deviation, *SE* standard error of mean, *LS* least squares

^a^The time to onset of syncopal symptoms/near-syncopal symptoms was defined as the duration (in seconds) from the initiation of the protocol-defined tilt-table test until syncopal symptoms/near-syncope (of sufficient severity that caused the patient to ask that the tilt table be returned to the horizontal position)

^b^For patients who completed the tilt-table test and did not achieve onset of syncopal symptoms/near-syncope, the time to onset was set to 2700 s

^c^LS Mean, standard error, difference in LS mean (midodrine HCl–placebo) and *p* value were based on type III sum of squares from an ANOVA model for time to onset of syncopal symptoms/near-syncopal symptoms, including treatment sequence (two levels), treatment (two levels), and treatment period (two levels) as fixed effects and subject-within-sequence as a random effect

The difference in LS means between the treatments was statistically significant (mean difference: 521.0 s, 95 % confidence interval: 124.2–971.7 s; *p* = 0.0131), thus meeting the primary endpoint. In total, six patients receiving midodrine and one patient receiving placebo did not have syncopal symptoms or a near-syncope event during the 45-min tilt-table test.

### Safety evaluation

No midodrine-related incidences of supine hypertension were recorded. Eight patients in the enrollment set experienced a total of 11 treatment-emergent adverse events (TEAEs) in Part A, all of which were mild or moderate in severity and none of which was considered by the investigator to be related to midodrine. The most frequent TEAEs were nausea and headache (two patients experiencing two events in both instances).

Two patients experienced a total of four TEAEs in Part B, all of which were mild or moderate in severity and none of which was considered by the investigator to be related to midodrine. One patient experienced a TEAE of back pain after receiving placebo, and one patient experienced three TEAEs (fatigue, flushing, and hot flush) after receiving midodrine. There were no notable concerns related to clinical laboratory evaluations, ECG results, or health status.

## Discussion

This study used a randomized-crossover design to assess the clinical benefit of midodrine in terms of symptom response compared with placebo in patients with symptomatic orthostatic hypotension. Symptoms were induced in a controlled inpatient setting using a 45-min tilt-table test that was performed at 1 h post-dose with midodrine or placebo. Treatment with midodrine provided a statistically significant increase in time to tilt-table-induced syncopal symptoms or near-syncope in patients with symptomatic orthostatic hypotension when compared with placebo.

Changes in blood pressure correlate with the frequency and severity of syncopal symptoms in patients with orthostatic hypotension, and this has been used as a surrogate measure of the effectiveness and clinical benefit of orthostatic hypotension treatments. Midodrine has previously been shown to increase blood pressure and vascular tone, which correlates with a reduction in frequency and severity of syncopal symptoms [11, 12]. Further studies were required to evaluate midodrine’s clinical benefit with regard to symptom response. In the present study, patients responding well to midodrine treatment (stable dose for at least 3 months) were enrolled. Midodrine-related blood pressure changes and improvement in OHSA scores in part A of the study served to identify those patients for whom midodrine provided a clear clinical benefit. Subsequent analyses during part B demonstrated the midodrine-related symptomatic improvement of these patients in a controlled clinical setting.

**Fig. 3:**
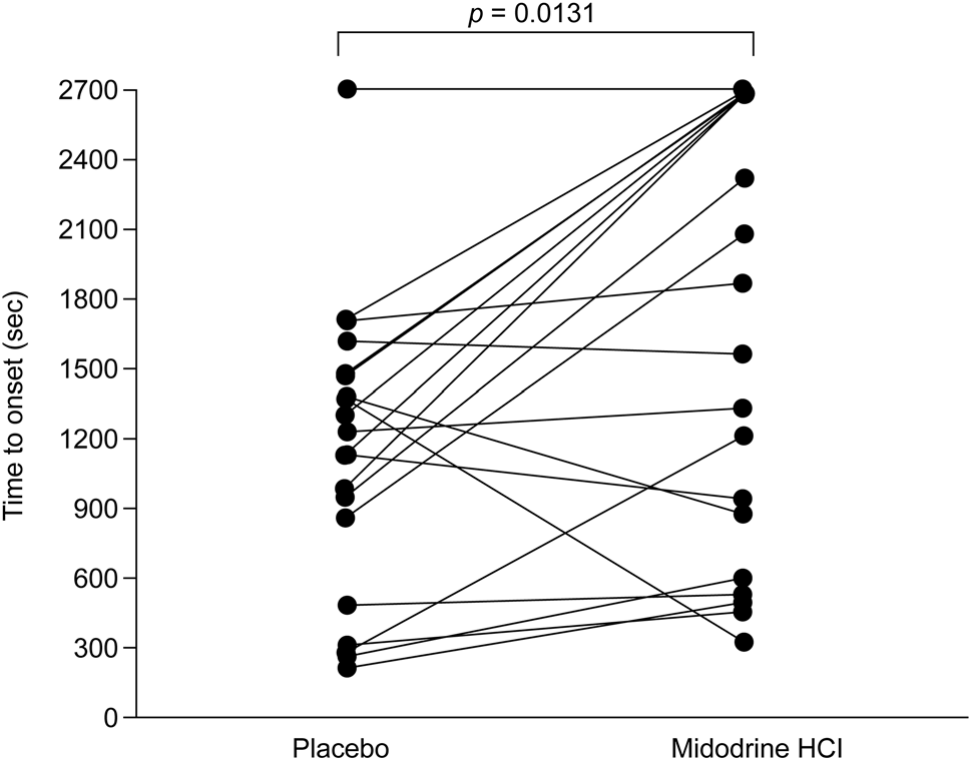
Time to onset of syncopal symptoms/near-syncope after initiation of a tilt-table test in patients receiving midodrine or placebo in the final analysis set The time to onset of syncopal symptoms/near-syncopal symptoms was defined as the duration (in seconds) from the initiation of the protocol-defined tilt-table test until syncopal symptoms/near-syncope (of sufficient severity that caused the patient to ask that the tilt table be returned to the horizontal position). For patients who completed the tilt-table test and did not achieve onset of syncopal symptoms/near-syncope, the time to onset was set to 2700 s. The *p* value was based on type III sum of squares from an ANOVA (analysis of variance) model for time to onset of syncopal symptoms/near-syncopal symptoms, including treatment sequence (two levels), treatment (two levels), and treatment period (two levels) as fixed effects and subject-within-sequence as a random effect

A history of severe orthostatic hypotension was one of the primary criteria for enrolment in the study, and according to the selection process for part B of the study, the patients were required to exhibit a >4 point decrease in their OHSA score and an acute drop in orthostatic blood pressure on withdrawal of midodrine, confirming the severity of their hypotension. Although a mean tilt time of 1105 s with placebo may seem long for patients with this disease, the majority of patients were being managed with multimodal treatment. Within this trial, patients were able to maintain all other treatments, such as fludrocortisone, which may have influenced the seemingly long time to response in the placebo group. Interestingly, as shown in Fig. 3, the patients who had a longer tilt time with placebo seemed to exhibit greater improvement with midodrine than those patients with shorter placebo tilt times of <10 min. As this patient population had been under treatment for at least 3 months and the study was focussed on evaluating the symptomatic response in patients with a confirmed midodrine-related clinical benefit, an expectation of similar results for typical patients with immediate-onset (<1–2 min) symptomatic orthostatic hypotension is unlikely.

Midodrine has been associated with supine hypertension, and there is no clinical trial experience in patients with supine blood pressure greater than 180/110 mmHg. Systolic pressure of about 200 mmHg was observed in 13.4 % of patients taking 10 mg midodrine, seemingly associated with a mean pre-treatment supine systolic blood pressure of 170 mmHg. Midodrine is not recommended for use in these patients, and to ensure the safety of patients enrolled in the study, the cut-off value for supine blood pressure (≥180/110 mmHg) was applied as an exclusion criterion. As only patients who had been on a stable dose for at least 3 months were included, the toleration of midodrine in the population was likely to be quite high. No incidences of supine hypertension were reported, none of the adverse events reported were considered to be related to midodrine, and no new safety signals were identified.

The primary diseases underlying orthostatic hypotension include Parkinson’s disease, pure autonomic failure, and multiple system atrophy, although non-neurogenic causes are more common and may be related to dehydration, drug therapy, and cardiovascular abnormalities [16]. Many patients with orthostatic hypotension have comorbidities related to autonomic nervous system dysfunction, such as cognitive impairment and diabetes mellitus [17]. These comorbidities display intra- and inter-day variations in intensity and could impact the assessment of the efficacy of midodrine versus placebo. In this study, the enrolment criteria were related primarily to the history and assessed severity of orthostatic hypertension and not the underlying diagnosis. As a result, the patient population included patients who were diagnosed with orthostatic hypotension at a younger age, who, thus, may have otherwise been excluded from previous phase 3 clinical trials; this included patients with depression, diabetes, Ehlers-Danlos syndrome, hypothyroidism and cancer, as well as patients with primary dysautonomias. Patients with orthostatic hypotension are usually elderly, with prevalence increasing with age (10–30 % in the elderly), and study enrollments usually reflect this [18]. However, studies have also shown a prevalence of 5.1–6.2 % in middle-aged patients [19, 20], with increased mortality risk in patients with orthostatic hypotension younger than 42 years [21]. By evaluating a real-world population in which the patients’ ages ranged between 18 and 78, the benefit provided by clinical application of midodrine with regard to patients’ symptoms and individual responses could be demonstrated in a controlled environment for a heterogeneous population, with a wide age distribution and a range of associated diagnoses.

Although the use of tilt-testing as implemented in this study is quite different from real-life situations, midodrine was highly efficacious in this controlled clinical setting. A previous midodrine study reported that improved hemodynamic responses to a tilt-table test were associated with reductions in the symptoms of hypotension and with improvements in health-related quality of life [22]. Midodrine is clearly beneficial in the studied environment, but more detailed analyses would give greater insight into the effect on the patients’ quality of life.

Developing effective therapies for orthostatic hypotension is vitally important as it greatly affects a patient’s day-to-day functioning: it can be associated with increased incidence of heart failure, cerebrovascular disease, myocardial infarction, and falls [19, 23, 24], and some studies suggest that it can also cause cognitive impairment [25]. Orthostatic hypotension is difficult to treat, particularly as orthostatic stress is not uniform throughout the day [1]. In addition, treatments that elevate standing blood pressure may cause problems, such as severe supine hypertension [26]. Many commonly recommended therapies used in the treatment of orthostatic hypotension have a limited evidence base and lack high-quality data from randomized, controlled clinical trials [27]. The results of this study are supportive of earlier investigations into the efficacy of midodrine treatment for orthostatic hypotension, which assessed a variety of dosing frequencies and concentrations [11, 12]; these randomized, placebo-controlled, double-blind studies reported that the proportions of patients experiencing an improvement in standing systolic blood pressure and a reduction in the major symptoms of orthostatic hypotension (dizziness or lightheadedness, and unsteadiness) were significantly larger after receiving midodrine than after receiving placebo.

The range of orthostatic symptom severity is extremely wide, and the same treatments are not suitable for all patients. Patients with mild symptoms can be well treated with intravascular volume repletion or support stockings, while patients with more severe symptoms require much more aggressive medical management [1]. In addition to the previously reported physiological effect of sustaining blood pressure in patients with symptomatic orthostatic hypotension [7, 11, 12], this study has shown that midodrine offers a clinical benefit with regard to its efficacy in prolonging symptom-free orthostasis. As a clinically effective and well-tolerated treatment for symptomatic orthostatic hypotension, midodrine is an important treatment option for those patients who are not adequately treated using simpler measures.
